# Revisiting the “Timed Up and Go” test: a 12-s cut-off can predict Hospitalization Associated Functional Decline in older adults

**DOI:** 10.1007/s11357-024-01280-3

**Published:** 2024-07-16

**Authors:** Orly Gatenio-Hefling, Roy Tzemah-Shahar, Kfir Asraf, Omer Dilian, Efrat Gil, Maayan Agmon

**Affiliations:** 1 Faculty of Health and Social Welfare, University of Haifa, Haifa, Israel; 2 Max Stern Yezreel Valley College, Emek Yezreel, Israel; 3 HaEmek Medical Center, Afula, Israel; 4 Faculty of Medicine, Technion Institute of Technology, Haifa, Israel

**Keywords:** Functional decline, Assessment, Mobility, Risk

## Abstract

Early detection of functional decline, a major risk among hospitalized older adults, can facilitate interventions that could significantly reduce it. We aimed to examine the contribution of the Timed Up and Go (TUG) test in predicting Hospitalization Associated Functional Decline (HAFD) among older adults, able to independently ambulate before admission. We used a cross-sectional study design; a total of 310 older adults (age ≥ 65) hospitalized in internal medicine wards between December 2018 and August 2020 were included; exclusion criteria were inability to ambulate, a diagnosis restricting mobility, hospitalization for end-of-life care, or impaired cognition. The Modified Barthel Index was used to assess HAFD; it was administered at admission to evaluate patients’ independence in activities of daily living 2 weeks prior hospitalization, and at discharge. The TUG test was performed on admission and to predict significant functional decline (defined by a reduction of three points or more in the Modified Barthel Index), while accounting for demographics, length of hospitalization, comorbidity burden (Charlson’s comorbidity index), and cognitive function (ALFI-MMSE). Participants were divided into three groups according to their TUG score—under or over a cut-off score of 12 s, or inability to complete the test. Adjusting for age, comorbidity, cognitive ability, and duration of hospitalization, the group that performed the test in less than 12 s showed no statistically significant change in the Modified Barthel Index, therefore no significant HAFD. The other groups showed a statistically significant decline in function. Risk for significant HAFD is currently underestimated in clinical settings, limited to subjective assessment, and underused in the context of implementing early interventions to prevent HAFD. The TUG may support screening for those at risk of hospitalizing-associated functional decline and could help identify patients suitable for preventative interventions.

**Supplementary Information:**

The online version contains supplementary material available at 10.1007/s11357-024-01280-3.

## Introduction

Hospitalization Associated Functional Decline (HAFD) in older adults has been recognized as a major phenomenon with significant consequences such as slower recovery, higher rates of nursing home placement, and increased morbidity and mortality [1–4]. Functional decline, defined as a reduction in functional autonomy and an increase in disability [5], was previously linked with reduced mobility levels among older adults both in community and hospital settings [6, 7]. Moreover, functional decline was found to be associated with risk of falls or a consequence of low mobility abilities [8]. Many factors were previously associated with a risk to develop HAFD, most of which can be divided into personal or environmental factors [9, 10]. However, there is currently no objective test that can directly estimate personal risk for HAFD. Having a simple, easy to administer, objective tool for early identification of individuals at greater risk of functional decline may support implementation of early, hospitalization-specific, mobility programs aiming to reduce older adults’ functional decline during hospital stays [4] or otherwise support proper utilization of hospital resources.

Function can be measured objectively or subjectively [11, 12]. Subjective assessment, such as The Activities of Daily Living Questionnaire [13], is based on a subjective rating of the patient’s ability, while objective assessment of function involves tasks that requires demonstration of the ability to walk, get up from a chair, maintain balance while performing a task, and so on (e.g., Short Physical Performance Battery, 6-min walk) [14], offering a more reliable metric of function [15]. Functional decline can be estimated using different tests or clinical tools [16]; one such tool used in hospital settings is the Barthel Index [1, 4].

The Barthel Index includes 10 items relating to activities of daily living and mobility (e.g., bathing, dressing, chair transfer, stair climbing), rated by level of independence to perform each item [17, 18]. The Barthel Index is commonly performed based on a subjective report by the patient or family members [19]. The total score ranges from 0 to 100, and the minimum clinically important difference changes across cohorts, ranging from 1 to 10 points or more, depending on the reference population [20–22]. Among ambulating older adults, a 3 points reduction in the Barthel Index is considered a minimum clinically important difference [21]. Using simple and feasible functional measures, such as the Timed Up and Go (TUG) test, can provide a better assessment for HAFD risk by adding an objective dimension of mobility to currently available predictors [23].

The TUG test, an objective assessment of mobility, is linked with an individual’s functional ability, including lower extremity muscular strength [24], aerobic capacity, and walking endurance [25]. The test, commonly used as a measure of mobility and for risk of falls assessment [26], is comprised by getting up from a chair, walking 3 m, turning, and returning to the starting seated position. The TUG test is extensively used in community-based facilities and is recommended by various geriatric societies both for assessing older adults’ mobility and as a screening tool for risk of falls [27, 28]. The TUG test was also found to be associated with other various impairments in older adults, such as frailty [29], severity of Parkinson’s disease [30], and functional decline [31]. The TUG test is also associated with cognition, as the test procedure requires adequate attention and executive function [32]; thus, it extends our ability to quantify an individual’s ability to participate in activities of daily living.

The TUG test has clear advantages relative to other mobility tests, such as the de Morton Mobility Index [33] and the Acute Care Mobility Assessment [34]: it is objective, takes a short time to complete, and is easy to administer [23]. The parameter of interest in the TUG test is time-to-completion. Different studies have used various TUG test clinical cut-off points, ranging from 13 to 32.6 s [35]. The Centers for Disease Control and Prevention (CDC) Stopping Elderly Accidents, Death and Injuries (STEADI) initiative suggests using a relatively rigorous cut-off point of 12 s to differentiate individuals at risk [36], suggesting that setting a higher performance bar is preferable. Moreover, although inability of hospitalized older adults to complete the TUG test is perceived as a hinderance to its usability, some studies show that this response can also be informative—for example, inability to complete the TUG test was found to be predictive of future falls [37] and of length of hospitalization [38].

Despite its use in community facilities, the TUG test is regarded as an objective measure for risk of fall by most practitioners [39], and thus is not commonly used for assessing functional ability in acute care hospital settings. Re-examining it as a tool for objective assessment of function would be beneficial, as HAFD has been shown to be modifiable through in-hospital interventions [1, 4, 40]. This study suggests expanding the existing model of the TUG test to include three categories (i.e., completion under 12 s, completion 12 s and over, and not being able to complete the test); we aimed to examine the use of the TUG test, with these three objective categories, as a possible screening tool for HAFD risk in older hospitalized patients.

## Methods

### Setting

This observational longitudinal study is part of the HoPE-MOR (Hospitalization Process Effects on Mobilization Outcomes and Recovery) study, conducted in two internal medical units in two hospitals in northern Israel (Ha’emek Medical Center and Bnei-Zion Medical Center). The study was approved by the ethics committee of the Faculty of Social Welfare and Health Sciences at University of Haifa, Israel (approval no. 324/17) and funded by the Israel Science Foundation (ISF 1216/17).

### Participants

Older adults (age ≥ 65 years), who were admitted in one of the two internal medicine units via the adjacent emergency department due to an acute condition or the exacerbation of a chronic condition, were screened for participation in the study. Inclusion criteria were speaking Hebrew, Russian, or Arabic and being able to walk at time of admission. Exclusion criteria were a diagnosis or a condition restricting walking ability (e.g., hip fracture, wheelchair user), hospitalization for end-of-life care, isolation due to resistant bacteria, and impaired cognition (Short Portable Mental Status Questionnaire score ≤ 5 or a score of > 4 in the 4AT Delirium Screening Tool) [41, 42]. Written consent was obtained from all participants prior to any study procedure.

### Study procedures

The TUG test was administered within 24 h of admission by research team members who were trained beforehand by one of the authors (a physical therapist) and periodically observed to ensure procedure fidelity. The participants were asked to stand up from a standard height chair (seat height approximately 46 cm, as per TUG test guidelines [43]), walk around a cone positioned at 3 m, and return to the seated position [23]. The test was demonstrated by a study team member prior to measurement and a single training attempt was allowed for familiarity with the test. A study team member remained in proximity to the participant to assure safety, without interfering with the procedure. Time to complete the test was recorded using a stopwatch; failure to complete was also recorded. The TUG test has a good test–retest reliability and excellent test–retest reliability and validity in similar population [44]. Functional decline was assessed using The Modified Barthel Index [45]; this version of the tool was chosen for its ability to detect more subtle changes in ability than the original version [46]. An individual’s subjective assessment for level of independence in 10 activities of daily living was rated, for a total score ranging from 0 (completely dependent) to 100 (completely independent). The tool was administered by research team members who were trained beforehand by one of the authors (a licensed nurse) and periodically observed to ensure procedure fidelity. The Modified Barthel Index administered at admission was used to evaluate patients’ independence in activities of daily living 2 weeks prior to administration. Administration at discharge provided quantification of HAFD. The Barthel Index has a good test–retest reliability and validity as a measure of physical dependency [47].

Demographic data including age, gender, and duration of hospitalization (days) were collected from medical records. The burden of chronic medical diagnosis was assessed using Charlson’s comorbidity index (CCI) [48] and was filled out by the research team within 24 h of admission; CCI refers to 19 health conditions divided into three categories of severity, with scores ranging from 0 to 37. Higher scores indicate higher risk of mortality. Cognitive function was assessed within 24 h of admission using the Adult Lifestyle and Function Interview—Mini-Mental State Examination (ALFI-MMSE) [49], a reliable and validated short version of the MMSE. This tool includes 22 items, with scores from 0 to 22, where lower scores indicate lower cognitive function; a score of 17 points or less in ALFI-MMSE is used to discriminate individuals with impaired cognition.

### Statistical analysis

Analyses were performed using either SPSS 27 (IBM SPSS Statistics, New York, US) or Jamovi (v. 2.4.11) [50]. Continuous data were presented as either mean ± SD or mean ± SE, as appropriate, and categorical data were presented as percentage and raw number of individuals. Differences in demographic variables were examined by Welch’s ANOVA with Games-Howell tests for post hoc comparisons, the Kruskal–Wallis with Dwass-Steel-Critchlow-Fligner (DSCF) tests for post hoc comparisons, and Pearson’s chi-squared tests, with post hoc comparisons conducted via adjusted standardized residuals. Games-Howell and DSCF tests control for familywise type I error, and false discovery rate adjustment was used for the post hoc comparisons for Pearson’s chi-squared tests. To estimate exact *p*-values for Pearson’s chi-squared tests, the Monte Carlo sampling method (200,000 samples) was applied to compute *p*-values. As Modified Barthel Index scores are count data, a mixed Poisson model was used. The model included random intercepts for participants and hospitals as random variables, to account for random variability.

## Results

Data collection was initiated in December 2018 and terminated in August 2020; a total of 2615 older adults were admitted to both units, and a total of 310 participants (age 77 ± 7) were recruited to this study. A flow chart with full procedures and reasons for exclusion is provided as supplementary Fig. S1. Included participants were divided into three groups according to their TUG test results, based on the 12 s cut-off point set by the CDC [36] or inability to complete the test altogether. Group 1 included 46 participants scoring TUG < 12 s; group 2 included 165 participants scoring TUG ≥ 12 s, and the remaining 99 participants were included in group 3, being unable to complete the TUG test. Table 1 presents participants’ demographics as well as covariates data; ALFI-MMSE scores (global cognitive function) were significantly different between the groups, with group 1 exhibiting the highest scores. Participants allocated to group 1 were significantly younger than patients from groups 2 and 3. No differences were found between the groups regarding sex and CCI (comorbidity index).

**Table 1:** Demographic and covariates data

		Mean ± SD (Median) / Frequencies, n				Post-hoc comparisons		
Variable (range)	Full sample (*n*=310)	TUG < 12 (*n*=46)	TUG ≥ 12 (*n*=165)	TUG incompletion (*n*=99)	Statistic	TUG < 12 vs TUG ≥ 12	TUG < 12 vs TUG incompletion	TUG ≥ 12 vs TUG incompletion
Age	77.08 ± 7.01	74.63 ± 6.43	77.33 ± 7.03	77.80 ± 7.05	F _Welch (2, 124.07)_ = 3.93, *p*=.022	t_(77.64)_= –2.46, *p*=.041	t_(95.65)_= 2.68, *p*=.023	t_(205.98)_= 0.52, *p*=.856
% of Females	47.10 (*n*=146)	36.96 (*n*=17)	47.88 (*n*=79)	50.51 (*n*=50)	χ^2^_(2)_ = 2.40, *p*=.301			
CCI (0–37)	1.90 ± 1.75 (1)	1.45 ± 1.57 (1)	1.89 ± 1.82 (1)	2.14 ± 1.68 (2)	H _(2)_ = 6.24, *p*=.044	W=2.01, *p*=.330	W=-3.53, *p*=.033	W=-2.13, *p*=.286
ALFI- MMSE (0–22)	18.59 ± 3.04 (19)	19.84 ± 2.59 (21)	18.64 ± 2.90 (19)	17.83 ± 3.31 (18)	H _(2)_ = 13.80, *p*=.001	W=-3.97, *p*=.013	W=-5.00, *p*=.001	W=2.49, *p*=.180
TUG (sec)	20.50 ± 9.41 (18)	10.47 ± 1.38 (11)	23.29 ± 8.76 (21)	-	H _(1)_ = 107.49, *p* < .001			
Duration of hospitalization (days)	5.92 ± 5.47 (4)	4.08 ± 1.83 (4)	5.92 ± 6.09 (4)	6.79 ± 5.30 (6)	H _(2)_ = 13.79, *p*=.001	W=2.23, *p*=.255	W=-5.00, *p*=.001	W=-3.82, *p*=.018
						TUG < 12	TUG ≥ 12	TUG incompletion
% of ALFI- MMSE < 17	23.53 (*n*=68)	8.89 (*n*=4)	23.13 (*n*=37)	32.14 (*n*=27)	χ^2^_(2)_ = 8.83, *p*=.012	χ^2^_(1)_ = 6.25, *p*=.012	χ^2^_(1)_ = 0.04, *p*=.841	χ^2^_(1)_ = 4.84, *p*=.027

Total sample size is below 310 in some measures due to missing data. Post-hoc comparisons are Games-Howell for Welch's ANOVA, DSCF (Dwass-Steel-Critchlow-Fligner test; W statistic) for Kruskal-Wallis (H statistic) and adjusted standardized residuals for Pearson's chi-squared tests. Post hoc for Pearson's chi-squared tests are FDR adjusted for multiple comparisons, with the threshold of significance for % of ALFI- MMSE < 17 is *p* <.033. *CCI* Charlson Comorbidity Index (higher is worse). *ALFI-MMSE* Mini–mental state examination conducted through telephone (Higher is better). *TUG* Time up and go (Higher is worse)

**Fig. 1:**
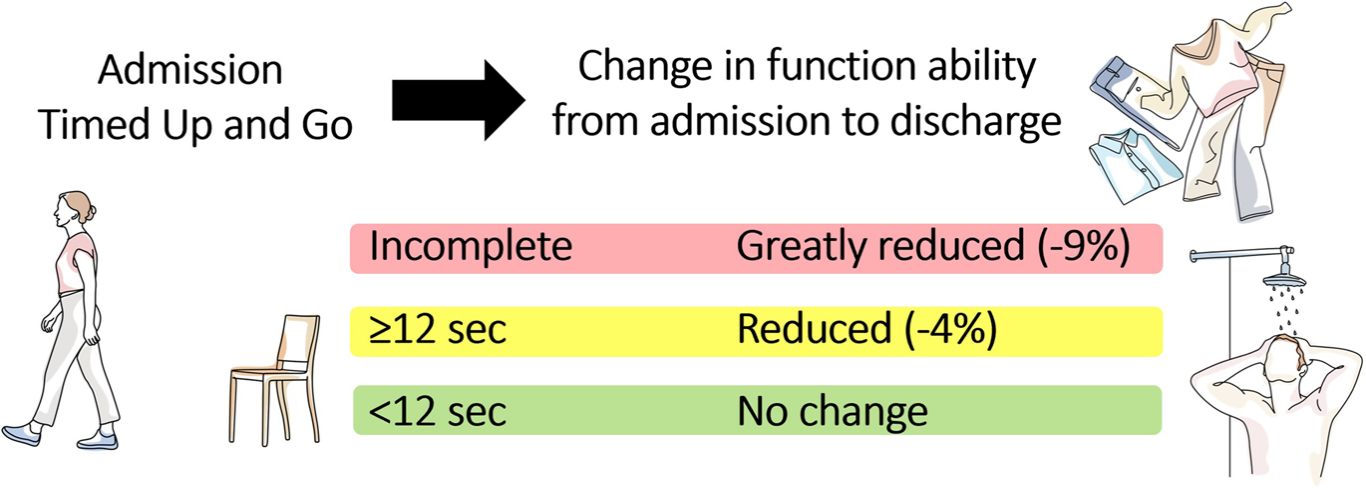
Visual summary of the main results

Table 2 presents the cohort descriptive statistics; Tables 3 and 4 present the mixed Poisson model results, comparing the model-adjusted Modified Barthel Index scores at each time point while controlling for selected covariates. Note that while group 1 showed no clinically significant change in Modified Barthel Index scores, the other two groups demonstrate a significant functional decline, shown by ΔBarthel Index of − 4.03 and − 8.79 respectively. Figure 1 presents a visual summary of the main results (note that “greatly reduced” refers to the magnitude of change in ΔBarthel Index).

**Table 2:** Descriptives of Barthel Index score, and measured change at admission vs. discharge

	Barthel Index – Mean ± SD (median; range)		
Barthel Index/group	TUG < 12 (*n* = 46)	TUG ≥ 12 (*n* = 165)	TUG incompletion (*n* = 99)
Admission^1^	99.78 ± 1.47 (100; 90–100)	95.23 ± 8.35 (100; 63–100)	81.64 ± 20.77 (90; 18–100)
Discharge	99.93 ± 0.33 (100; 98–100)	91.20 ± 15.26 (100; 22–100)	72.85 ± 28.41 (81; 4–100)
∆Barthel Index	+ 0.15	− 4.03	− 8.79

Barthel Index = modified Barthel Index score (higher is better). ^1^On admission, Barthel Index score were reported subjectively for level of independence 2 weeks prior to hospitalization

**Table 3:** Mixed Poisson model within-groups comparisons of Modified Barthel Index score change over time

	Barthel Index – Mean ± SE			
Group/Barthel Index	Admission^1,2^ (90% C.I.)	Discharge^2^ (90% C.I.)	*Z*	*p*
TUG < 12 s	94.01 ± 3.07 (89.09, 99.21)	94.39 ± 3.47 (88.85, 100)	-0.16	0.866
TUG ≥ 12 s	91.64 ± 1.74 (88.82, 94.56)	87.96 ± 2.20 (84.41, 91.66)	2.70	0.006
TUG incompletion	76.92 ± 1.86 (73.92, 80.05)	69.66 ± 2.04 (66.38, 73.10)	4.90	< 0.001

Barthel Index score range 0–100; higher score is better. ^1^On admission, Barthel Index score were reported subjectively for level of independence 2 weeks prior to hospitalization. ^2^Model-adjusted scores

**Table 4:** Mixed Poisson model between-groups comparisons of Modified Barthel Index score change over time

	Barthel Index^2^ – Mean + SE				Post hoc comparisons		
Group/Barthel Index	TUG < 12 s	TUG ≥ 12 s	TUG incompletion	*χ*^2^_(2)_	TUG < 12 vs TUG ≥ 12	TUG < 12 vs TUG incompletion	TUG ≥ 12 vs TUG incompletion
Admission^1^	94.01 ± 3.07	91.64 ± 1.74	76.92 ± 1.86	24.31 *p* < 0.001	Z = 0.75 *p* = 0.447	Z = 5.34 *p* < 0.001	Z = 6.53 *p* < 0.001
Discharge	94.39 ± 3.47	87.96 ± 2.20	69.66 ± 2.04	46.35 *p* < 0.001	Z = 2.07 *p* = 0.037	Z = 8.00 *p* < 0.001	Z = 8.62 *p* < 0.001

The Barthel Index score range 0–100; higher score is better. ^1^On admission, Barthel Index score were reported subjectively for level of independence 2 weeks prior to hospitalization. ^2^Model-adjusted scores

The full model for the change in Modified Barthel Index score explained 80.17% of the variance [R^2^_conditional_; LRT *χ*^2^_(13)_ = 1224.11, *p* < 0.001], and the fixed factors explained 30.62% of the variance in the full model [R^2^_margianl_; LRT *χ*^2^_(9)_ = 131.39, *p* < 0.001]. The Group * Time interaction was statistically significant [*χ*^2^_(2)_ = 14.45, *p* < 0.001], and post hoc testing revealed that groups 1 and 2 had significantly higher Modified Barthel Index scores comparing to group 3 at admission and discharge; this difference was more pronounced at discharge, mainly due to the greater reduction in group 3 (see Tables 3 and 4). On admission, no differences were found between TUG completers (groups 1 and 2); however, due to significant reduction in the Modified Barthel Index scores in group 2 at discharge, significant difference was found between groups 1 and 2. The model, adjusted for age, comorbidity (CCI), cognitive state (ALFI-MMSE) and length of hospitalization, suggests a ΔBarthel Index of − 3.68 for the TUG ≥ 12 group (decrease of 4.02%; mean ratio = 0.959, 95% C.I. = 0.931, 0.988) and − 7.26 for the TUG incompletion group (decrease of 9.44%; mean ratio = 0.905, 95% C.I. = 0.870, 0.942). This statistically significant reduction in the Modified Barthel Index, observed in groups 2 and 3, indicates a clinically significant functional decline.

## Discussion

This study aimed to examine TUG as a possible screening tool for risk of functional decline in older hospitalized patients. The findings demonstrate that categorizing hospitalized ambulating older adults based on their TUG performance can add predictive information on their potential to experience HAFD. These findings suggest that the 12 s cut-off set by the CDC, as well as an incompletion category, can identify individuals at risk for significant HAFD and facilitate implementation of designated interventions aiming to maintain functional capacity; these findings remain significant while adjusting for age, comorbidity, cognitive state, and length of hospitalization of the participant.

Incompletion of the TUG test is a relatively underused and understudied metric, previously reported to predict falls in the context of acute care [37]. This novel use of the inability to complete TUG test among participants who were previously ambulatory, for prediction of significant functional decline, expands the potential contribution of the TUG test in patient screening. Differentiating between hospitalized patients at greater risk for functional decline is essential for best care practices; administrating the TUG test is simple, requires minimal resources, and provided an objective screening tool for risk of functional decline. Moreover, the chosen 12 s cut-off, more demanding than the commonly used 13.5 s [35], sets a relevant clinically valuable marker for the use of the TUG test for assessing risk of functional decline, supporting its use for promotion of early, in hospital, mobility interventions [51], or interventions following discharge [52]. Moreover, identifying individuals at greater risk for a significant HAFD may facilitate better utilization of health resources during and following hospitalization [53].

Significant differences in cognitive function and length of hospitalization between the groups were observed (Table 1). Indeed, cognitive status was previously associated with the TUG test [54], and length of hospitalization stay is also linked with greater HAFD [55]. Thus, we accounted for these variables in the statistical model that demonstrates the significant predictive value of the TUG test even after controlling them. Of note, patients with delirium or severe cognitive impairment were excluded.

Previous studies have demonstrated that various interventions during hospitalization, such as engaging patients in daily life activities and physical activities [2], can have a mediating effect on functional decline. Moreover, an individualized, multicomponent exercise intervention may reverse functional decline associated with hospitalization [1]. Patients identified as being at risk for functional decline should be the target population for such interventions and, comparably, when identifying patients with high mobility capacity and lower risk for functional decline, it may be advisable to refrain from limiting their mobility during hospitalization due to safety concerns [56], or use alternatives intending to promote mobility while maintaining safety.

Limitations: This study was conducted in two hospitals with a sample of relatively high functioning older adults; thus, its generalizability is limited. Future studies may examine the use of the three TUG categories for the prediction of functional decline in various samples of older inpatients to establish generalizability. Future studies are encouraged to use objective measures of activities of daily living, which may offer a broader perspective for function, thus expanding our understanding of the relationships between TUG test results and functional decline.

## Conclusions and implications

This study has established the TUG test as a feasible screening tool for HAFD in older adults; by using the suggested categories, healthcare professionals can differentiate individuals at greater risk for functional decline. Older adults unable to successfully complete the TUG test in under 12 s or those unable to complete it altogether show significant functional decline following hospitalization, and thus may require early mobilization intervention effort, while hospitalized or immediately post-discharge, that may counteract the negative impact of acute hospitalization. Furthermore, where needed, additional resources (e.g., hospital-based physical therapy, informative guidance provided to accompanying family members) should be allocated to those at risk.

## Supplementary Information

Below is the link to the electronic supplementary material.

Supplementary file1 (JPG 90 KB)

## Data Availability

The data that support the findings of this study are available from the corresponding author, MA, upon request.

## Abbreviations

HAFD: Hospitalization Associated Functional Decline
TUG: Timed Up and Go
CDC: Centers for Disease Control and Prevention
STEADI: Stopping Elderly Accidents, Death and Injuries
CCI: Charlson’s comorbidity index
ALFI-MMSE: Adult Lifestyle and Function Interview—Mini-Mental State Examination
DSCF: Dwass-Steel-Critchlow-Fligner
