# Supplementary figures and images for: Revisiting the “Timed Up and Go” test: a 12-s cut-off can predict Hospitalization Associated Functional Decline in older adults
Source: GeroScience. 2024 Jul 16;47(1):1039–48. doi: 10.1007/s11357-024-01280-3 (PMC11872843; doi:10.1007/s11357-024-01280-3)

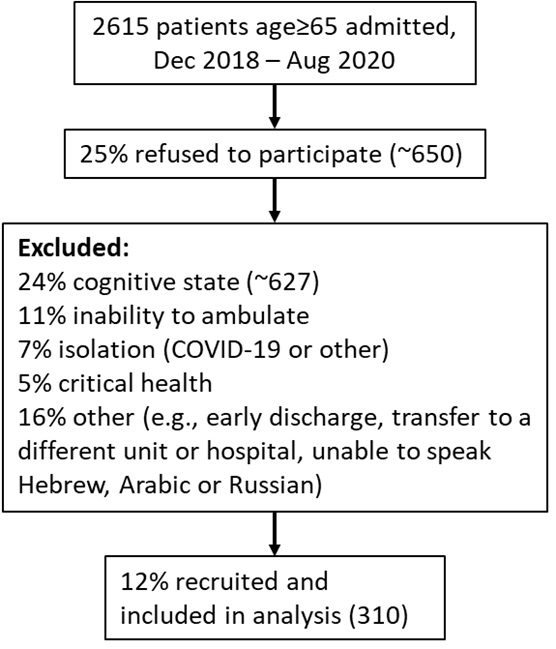

Supplement: Supplementary file 1 — Supplementary file1 (JPG 90 KB) [file 11357_2024_1280_MOESM1_ESM.jpg]
